# Soluble Oncoimmunome Signatures Predict Muscle Mass Response to Enriched Immunonutrition in Cancer Patients: Subanalysis of a Multicenter Randomized Clinical Trial

**DOI:** 10.3390/nu17152421

**Published:** 2025-07-24

**Authors:** Sara Cuesta-Sancho, Juan José López Gomez, Pedro Pablo García-Luna, David Primo, Antonio J. Martínez-Ortega, Olatz Izaola, Tamara Casañas, Alicia Calleja, David Bernardo, Daniel de Luis

**Affiliations:** 1 Mucosal Immunology Lab, Department of Paediatrics and Immunology, University of Valladolid, 47003 Valladolid, Spain; d.bernardo.ordiz@gmail.com; 2 Health Research Institute of Valladolid (IBioVALL), 47010 Valladolid, Spain; jlopezgo@saludcastillayleon.es (J.J.L.G.); dprimoma@saludcastillayleon.es (D.P.); oizaolaj@saludcastillayleon.es (O.I.); 3 Servicio de Endocrinología y Nutrición, Hospital Clínico Universitario de Valladolid, 47003 Valladolid, Spain; 4 Centro de Investigación de Endocrinología y Nutrición, Facultad de medicina, Universidad de Valladolid, 47003 Valladolid, Spain; 5 Servicio de Endocrinología y Nutrición, Hospital Virgen del Rocio, 41013 Sevilla, Spain; garcialunapp@yahoo.es (P.P.G.-L.); ajesus.martinez.sspa@juntadeandalucia.es (A.J.M.-O.); 6 Nutrimed Clinical Nutrition Medical Department, 35002 Las Palmas de Gran Canaria, Spain; t.casanas@nutrimedhealth.com (T.C.); a.calleja@nutrimedhealth.com (A.C.); 7 Centro de Investigaciones Biomédicas en Red de Enfermedades Infecciosas (CIBERINFEC), 28003 Madrid, Spain

**Keywords:** cancer, immunomodulation, precision nutrition, muscle mass, oral nutritional supplementation

## Abstract

Background/Objectives: Enriched oral nutritional supplementation (ONS) has been shown to increase muscle mass in cancer patients. This study aims to identify the immunomodulatory effects and predictive biomarkers associated with this intervention. Methods: The soluble levels of 92 immune- and oncology-related mediators were determined before and after an intervention (8 weeks) in 28 patients with cancer receiving either a standard (n = 14) or an enriched ONS (n = 14) using the Olink proteomics analysis pipeline (Olink^®^ Target 96 Immuno-Oncology panel (Uppsala, Sweden)) Results: Patients receiving enriched ONS experienced an average weight gain of 1.4 kg and a muscle mass increase of 2.2 kg after 8 weeks, both statistically significant (*p* < 0.05), while no such improvements were observed in the standard ONS group. Inflammatory markers TRAIL and LAMP3 were significantly reduced, along with an increase in Gal-1, suggesting lower inflammation and enhanced myogenic differentiation. However, patients who failed to gain muscle mass with the enriched formula showed a more aggressive inflammatory profile, characterized by higher serum levels of soluble MUC16, ARG, and IL12RB1. Interestingly, muscle mass gain could be predicted before the intervention, as responders had lower baseline levels of PGF, CD28, and IL12RB1. These differences were specific to recipients of the enriched ONS, confirming its immunomodulatory effects. Conclusions: Our findings support the use of enriched oral nutritional supplementation (ONS) as an effective strategy not only to enhance caloric and protein intake but also to promote anabolism and preserve muscle mass in cancer patients. The identification of immune profiles suggests that specific biomarkers could be used to predict which patients will benefit most from this type of intervention. This may allow for the implementation of personalized immunonutrition strategies that optimize resource allocation and improve clinical outcomes, particularly in vulnerable populations at risk of cachexia.

## 1. Introduction

Disease-related malnutrition (DRM) is prevalent among cancer patients and is driven by a complex interplay between tumor–host interactions and the adverse effects of anti-cancer therapies [1,2]. This systemic inflammatory response induces profound metabolic disturbances, including insulin resistance, enhanced lipolysis, and increased lipid oxidation. Consequently, patients experience fat loss, heightened protein catabolism, and muscle mass depletion accompanied by an increased production of acute-phase proteins. These cytokine-mediated metabolic alterations can impair the effectiveness of nutritional interventions [3].

To address DRM, oral nutritional supplementation (ONS) for cancer patients are typically enriched with high-quality protein, calories, and specific nutrients. Supplementation with eicosapentaenoic acid (EPA) and docosahexaenoic acid (DHA) has been associated with improvements in body weight, muscle mass, and improved energy and protein intake [4]. Additionally, amino acid supplementation, such as leucine, can enhance the patient’s anabolic capacity, while β-glucans, which are polysaccharides with immunoregulatory properties, have been associated with reduced levels of proinflammatory cytokines in cancer patients [5]. This targeted nutritional strategy is specifically called immunonutrition [6].

In this context, in a previous randomized, double-blind, multicenter clinical trial conducted by our group [7], cancer patients were randomized to either receive (i) specific high-calorie, high-protein oral nutritional supplements (ONS) enriched with leucine, EPA, DHA, extra-virgin olive oil (EVOO), and β-glucans (Alisenoc); or (ii) an isocaloric, iso-nitrogenous standard ONS (control). After eight weeks, only patients receiving enriched ONS displayed an increase in muscle mass, as measured by bioimpedance analysis (BIA). Previous studies have proven that enriched ONS helps cancer patients meet their nutritional requirements, especially those in more compromised clinical conditions, with high adherence, good tolerance, and positive acceptance [4]. These benefits translate into improved nutritional status, body composition, and functional outcomes [4]. However, the specific mechanism through which enriched ONS elicits its beneficial effects remains unclear.

The immunomodulatory potential of enriched oral nutritional supplementation (ONS) in cancer-related malnutrition, as demonstrated in our study, aligns with emerging evidence on how systemic inflammation and metabolic stress affect nutritional responses during periods of physiological disruption. Notably, the COVID-19 pandemic provided a unique context in which lifestyle changes and healthcare delivery modifications were shown to significantly impact the management of metabolic diseases such as type 2 diabetes, reinforcing the importance of individualized, resilient care pathways [7]. Furthermore, dietary behaviors during the pandemic were associated with altered nutrient intake and immune status, highlighting the bidirectional relationship between inflammation, immune mediators, and nutritional strategies [8].

Building from that, we hereby aimed to investigate whether the immune system could be responsible, at least to some extent, for the observed effects of such intervention on muscle mass. To this end, the primary aim of this study was to investigate the immunomodulatory effects of enriched oral nutritional supplementation (ONS) in cancer patients with DRM. The secondary aims were to identify soluble immune biomarkers associated with muscle mass response and to explore their potential as predictors of treatment efficacy. We analyzed the soluble oncoimmunome from these patients before and after the intervention. This proteomic approach provides a comprehensive understanding of how immunonutrition modulates systemic inflammation and muscle growth. Integrating these findings into clinical practice could therefore revolutionize the management of muscle-related DRM and other muscle-wasting conditions.

## 2. Materials and Methods

### 2.1. Patient Characteristics

A randomized, double-blind, parallel, multicenter clinical trial was conducted from March 2021 to May 2022 (NCT04184713). This study therefore provides a subanalysis related to the impact of an enriched ONS formula on the soluble oncoimmunome.

A total of 28 adult outpatients diagnosed with cancer were recruited. Tumor types included head and neck (n = 2), upper-gastrointestinal (n = 1), lower-gastrointestinal (n = 6), gynecological (n = 1), pancreatic (n = 2), pulmonary (n = 8), and urological (n = 4) tumors, as well as other less frequent malignancies such as thyroid and neuroendocrine tumors, lymphomas, sarcomas, and melanomas (n = 4). Eligible patients had either started or were about to start (within one month) antineoplastic treatment, including chemotherapy, immunotherapy, and/or radiotherapy (with or without prior surgery). Additionally, they were required to exhibit weight loss > 5% in the previous 6 months. Exclusion criteria included participation in other clinical trials, morbid obesity (body mass index [BMI] ≥ 40 kg/m^2^), previous or upcoming surgery as an oncology treatment, or the requirement for enteral/parenteral nutrition. Patients with refractory cachexia, severe infections or an infectious process requiring hospitalization, metabolic disorders such as diabetes mellitus or hyperglycemia due to corticosteroids, or who were were treated with insulin or with poor control (HbA1c > 8%) were also excluded. Other exclusion criteria included severe renal, cardiac, respiratory, or hepatic disease according to clinical criteria, severe active autoimmune diseases, or cognitive impairments such as dementia, moderate/severe mental illness, or decreased cognitive function. Finally, patients were further excluded if they had consumed ONS or artificial nutrition that could not be withdrawn at least 1 week before starting the study; taken food supplements or foods fortified in omega-3, arginine, leucine, hidroxy-methyl-butirate, or nucleotides in the previous month; refused to ingest the ONS; were pregnant or lactating women; or presented with an allergy or intolerance to any of the ingredients of the formulas under study. Patients who, at any time during the intervention, required nutritional treatment with enteral nutrition by tube or parenteral nutrition, who did not tolerate the product, or who ingested less than one unit of ONS per day were also withdrawn from the study.

Detailed patient selection criteria, the patient information sheet, informed consent, demographic variables, clinical variables, randomization, nutritional assessment and organoleptic evaluation of the nutritional supplement, adherence to nutritional treatment, and impact on dietary intake and tolerance to the supplement were described by Vidal Casariego et al. [4].

### 2.2. Dietary Intervention and Nutritional Evaluation

Following the ESPEN (European Society for Clinical Nutrition and Metabolism) recommendations for cancer patients, all patients received dietary recommendations to increase their energy and nutrient intake through their usual diets. In addition, patients were randomized to consume two bricks of either enriched ONS (Bi1 Alisenoc^®^, Nutrimed Clinical Nutrition, Las Palmas De Gran Canaria, Spain) (n = 14) or standard ONS (Bi1 Control 2.0 ^®^, Nutrimed Clinical Nutrition, Spain) (n = 14) daily for a period of 8 weeks. The compositions of both the enriched and the standard ONS are detailed in Table 1.

A three-day dietary assessment, incorporating a weekend day, was conducted following standardized instructions provided to the patient to ensure accurate completion. Food and beverage intake was quantified using a precision weighing scale or standard household measurement tools. A dietitian–nutritionist analyzed all dietary records utilizing the Dial version 3.0 software (Alce Ingeniería, Spain). This evaluation was carried out at both basal and end visit 2 timepoints. In both visits, stature (cm) was assessed using a standardized stadiometer, while body mass was measured with a digital scale (Omron, Los Angeles, CA, USA) under controlled conditions, with participants minimally clothed and barefoot. Body weight was recorded twice, and the mean value was used for analysis. Body mass index (BMI) was calculated as weight (kg) divided by height squared (m^2^). Total muscle mass was estimated via bioelectrical impedance analysis (BIA) using an EFG BIA 101 Anniversary device (Akern, Pisa, Italy), with a measurement precision of 50 g.

### 2.3. Biological Samples

Blood samples were collected from all participants before and after the ONS intervention. In all cases, blood was collected in gelose separator tubes and centrifuged at 800 g for 30 min at 4 °C (Fisherbrand™ GT2) to obtain serum samples, which were immediately aliquoted and cryopreserved (−80 °C) until analysis.

### 2.4. Soluble Oncoimmunome

Serum samples were analyzed with an Olink^®^ Target 96 Immuno-Oncology panel (Uppsala, Sweden) according to the manufacturer’s specifications. The levels of analyte-specific DNA amplicons for 92 soluble analytes were determined by Olink Analysis Service, following the requirements of ISO 17025. Testing of clinical trial samples was performed according to written SOPs and applicable GCP requirements. The results were quantified with an Olink Signature Q100, and the data were read out by the Olink NPX Signature software.

### 2.5. Statistical Analysis

The samples were subjected to a quality check of the incubation control and detection. Normalized protein expression (NPX) was expressed as Olink’s arbitrary unit, which is presented on a Log2 scale. It was calculated from Ct values, and data preprocessing (normalization) was performed to minimize both intra- and inter-assay variation. The NPX scale is inversely correlated with Ct.

Biomarker comparisons between groups and across timepoints were performed using either one-way or two-way ANOVA (for variables with a normal distribution) or the Kruskal–Wallis test (for non-normal distributions), following normality assessment via the Shapiro–Wilk test. Test selection was explicitly based on whether the variables met normality criteria. We did not apply equal variance tests separately but used appropriate non-parametric methods when normality was not met. Proteins with *p* values < 0.05 were considered significantly differentially expressed and were retained for further analysis

## 3. Results

### 3.1. Patients on Enriched ONS Improve Their Weight and Muscle Mass

Both nutritional interventions led to increased caloric, protein, and macronutrient intake. However, only patients under the enriched ONS treatment exhibited significant increases in leucine and EPA and DHA levels (Table 2). Importantly, these patients also demonstrated significant improvements in both body weight and muscle mass compared to those receiving the standard ONS (Table 3).

### 3.2. Each Patient Exhibits a Unique Oncoimmunome Fingerprint

To explore the mechanisms underlying the observed anabolic effects, the levels of 92 immune- and oncology-related mediators were determined before and after the intervention in 28 patients receiving either standard (n = 14) or enriched ONS (n = 14) using the Olink proteomics analysis platform (Table 4).

To facilitate interpretation, the following list presents all analytes in alphabetical order, each followed by its full molecular name as defined in the Olink panel:

ARG1: arginase 1; ANGPT2: angiopoietin-2; CASP_8: caspase-8; CCL17: C-C motif chemokine ligand 17; CCL20: C-C motif chemokine ligand 20; CCL23: C-C motif chemokine ligand 23; CCL3: C-C motif chemokine ligand 3; CD27: Cluster of Differentiation 27; CD5: cluster of differentiation 5; CD70: cluster of differentiation 70; CD83: cluster of differentiation 83; CSF_1: colony-stimulating factor 1; CX3CL1: C-X3-C motif chemokine ligand 1; CXCL10: C-X-C motif chemokine ligand 10; CXCL12: C-X-C motif chemokine ligand 12; CXCL13: C-X-C motif chemokine ligand 13; CXCL5: C-X-C motif chemokine ligand 5; DCN: decorin; Gal_1: galectin-1; GZMA: granzyme A; GZMB: granzyme B; HGF: hepatocyte growth factor; HO_1: heme oxygenase 1; ICOSLG: inducible T-cell costimulator ligand; IFN_gamma: interferon gamma; IL10: interleukin-10; IL12: interleukin-12; IL12RB1: interleukin-12 receptor subunit beta-1; IL13: interleukin-13; IL15: interleukin-15; IL4: interleukin-4; IL5: interleukin-5; KLRD1: killer cell lectin-like receptor D1; LAG3: lymphocyte activation gene 3; LAMP3: lysosomal-associated membrane protein 3; MICA/B: MHC class I polypeptide-related sequence A and B; MMP12: matrix metallopeptidase 12; MMP7: matrix metallopeptidase 7; NCR1: natural cytotoxicity-triggering receptor 1; PD_L1: programmed death-ligand 1; PD_L2: programmed death-ligand 2; PGF: placental growth factor; PTN: pleiotrophin; TNF: tumor necrosis factor; TNFRSF12A: TNF receptor superfamily member 12A; TNFRSF21: TNF receptor superfamily member 21; TNFRSF4: TNF receptor superfamily member 4; TRAIL: TNF-related apoptosis-inducing ligand; VEGFA: vascular endothelial growth factor A.

**Figure 1:**
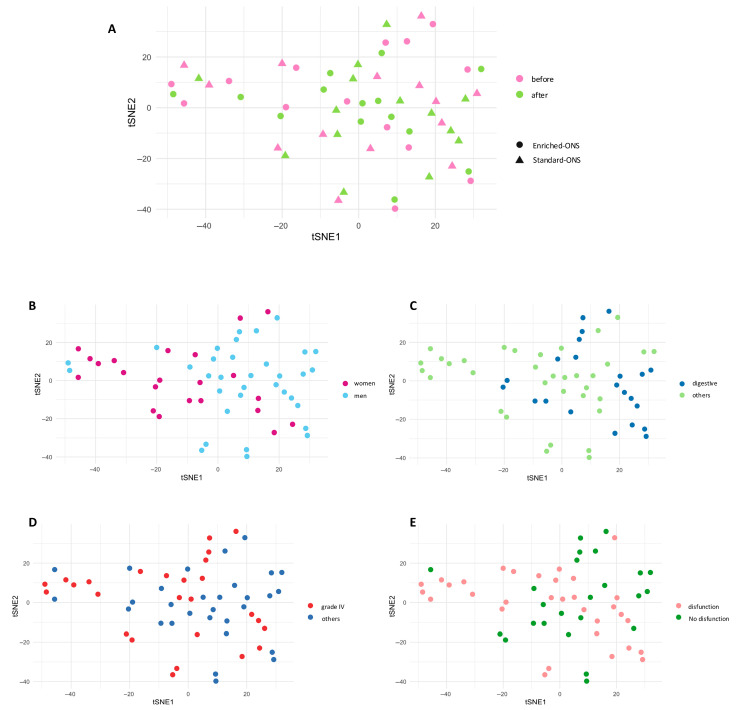
tSNE analysis of the observed variability. (**A**) tSNE analysis with the whole results from the whole soluble immunome from each patient, both before (pink) and after (green) the intervention with standard (▲) or enriched (●) oral nutritional supplementation (ONS). The analysis was also performed considering (**B**) the sex of each individual (blue for men, fuchsia for women); (**C**) the source of the cancer (blue for digestive, green for others); (**D**) the cancer stage (red for grade 4, blue for others); and (**E**) the presence (salmon) or absence (green) of dysfunction.

Given the high dimensionality of the dataset obtained through this approach, we first performed dimensionality reduction techniques to further explore the patient’s profile. Using the t-distributed stochastic neighbor embedding (tSNE) algorithm, this analysis revealed that patients did not cluster based on the ONS type or timepoint (Figure 1A). Notably, both paired samples from the same individual tended to cluster together, suggesting a strong patient-specific immunological signature. Further stratification by sex (Figure 1B), cancer type (Figure 1C), disease stage (Figure 1D), or functional capacity (Figure 1E) also failed to reveal any clear pattern. Hence, these findings suggest that the global soluble immunome is highly individualized and not influenced by clinical variables or intervention type.

### 3.3. Enriched ONS Reduces Inflammation and Enhances Myogenic Differentiation

**Figure 2:**
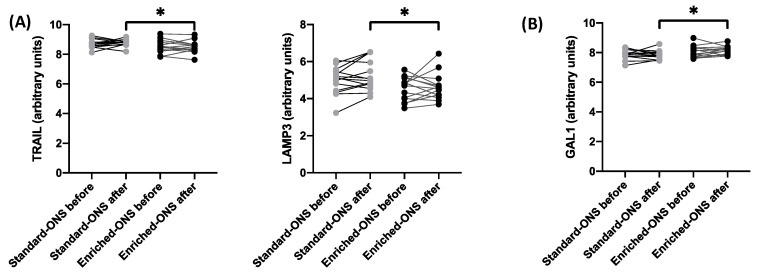
Non-responders to enriched ONS display a pro-inflammatory immune profileEffect of the intervention on the soluble levels from different immune mediators. The effect of the standard and the enriched oral nutritional supplement (ONS) on the levels of different immune mediators (in arbitrary units) was determined both before and after the intervention. TNF-related apoptosis-inducing ligand (TRAIL) and lysosome-associated membrane glycoprotein 3 (LAMP3), both associated with systemic inflammation, are shown in panel (**A**). Conversely, panel (**B**) displays elevated levels of galectin-1 (Gal-1), a protein implicated in myogenic differentiation and muscle regeneration. Paired *t*-test was applied to compare the effect of the intervention within each group, while unpaired *t*-test was performed for the comparison of the levels before or after the intervention on each type of ONS. *p*-value < 0.05 was considered as statistically significant (* *p* < 0.05).

Next, we performed an individual analysis for each of the 92 different oncoimmunome mediators. Patients receiving the enriched ONS exhibited significantly lower post-intervention levels of TNF-related apoptosis-inducing ligand (TRAIL) and lysosome-associated membrane glycoprotein 3 (LAMP3), both associated with systemic inflammation (Figure 2A). Conversely, these patients showed elevated levels of galectin-1 (Gal-1), a protein implicated in myogenic differentiation and muscle regeneration (Figure 2B). Together, these findings suggest that enriched ONS supplementation may exert dual effects by mitigating inflammation and promoting muscle growth and repair.

**Figure 3:**
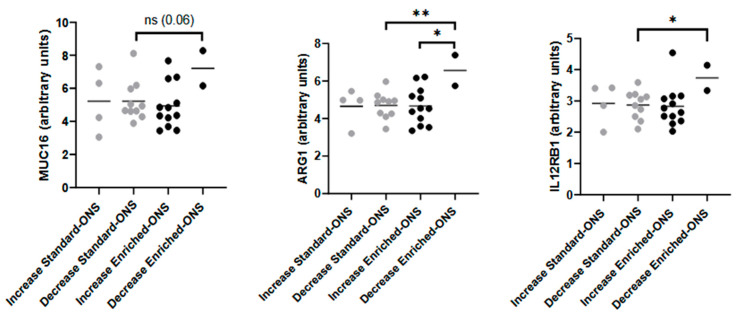
Soluble immunome analysis based on the increased or decreased muscle mass following the intervention. The effect of the standard and the enriched oral nutritional supplement (ONS) on the levels of different immune mediators (in arbitrary units) was determined based on the increase or decrease of the muscle mass following the intervention. One-Way ANOVA and subsequent unpaired *t*-test was applied. *p*-value < 0.05 was considered as statistically significant (* *p* < 0.05; ** *p* < 0.01, ns: not significant).

To investigate interindividual variability in response to enriched ONS, we stratified patients based on muscle mass changes following ONS intervention. Among those receiving the enriched ONS, individuals who did not gain muscle exhibited higher levels of soluble mucin 16 (MUC16), arginase 1 (ARG1), and interleukin 12 receptor beta 1 subunit (IL12RB1) compared to their counterparts receiving the standard ONS (Figure 3). Therefore, these findings suggest that specific immune profiles may influence individual responses to nutritional intervention, potentially identifying patients less likely to benefit from enriched ONS.

### 3.4. Baseline Immune Signature Predicts Muscle Mass Response

**Figure 4:**
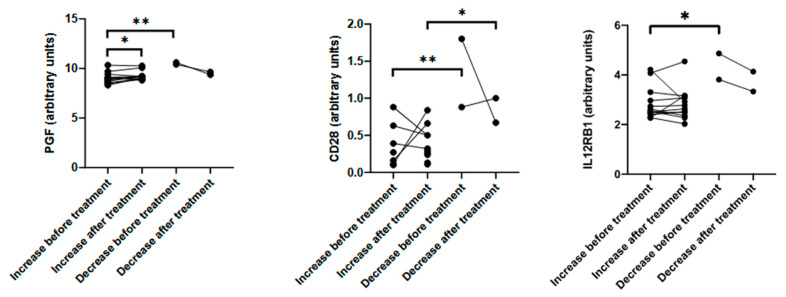
Immune dynamics of the immune mediators in patients treated with the enriched oral nutritional supplementation. The effect of the enriched oral nutritional supplement (ONS) on the levels of different immune mediators (in arbitrary units) was determined both before and after the intervention, in patients with an increase or a decrease of their muscle mass following the ONS. Paired or unpaired t-test was applied accordingly. *p*-value < 0.05 was considered as statistically significant (* *p* < 0.05; ** *p* < 0.01).

Having proven the presence of immune differences between patients who gained muscle mass and those who did not, we assessed whether these differences were already present before the intervention. Interestingly, patients who failed to gain muscle mass exhibited higher levels of soluble placenta growth factor (PGF), CD28, and beta 1 receptor for IL12/23 (IL12RB1) before the intervention (Figure 4). These findings suggest that specific immune signatures may serve as early biomarkers of response to immunonutrition, supporting the development of personalized nutritional strategies in cancer-related malnutrition.

## 4. Discussion

In this study, we provide, for the first time and to the best of our knowledge, a mechanism by which enriched ONS promotes muscle mass gain in cancer patients with DRM. Moreover, we have identified a panel of baseline biomarkers that may predict individual responses to enriched ONS, highlighting its potential as a tool for precision medicine in immunonutrition.

Our findings highlight the high degree of interindividual variability in the immune landscape of cancer patients. Dimensionality reduction techniques revealed no clustering based on ONS type, cancer characteristics, or functional status, underscoring the complexity and heterogeneity of the immune response in this population, particularly in relation to nutritional interventions. Indeed, despite the use of dimensionality reduction techniques, no distinct immune signature could be associated either with the type intervention. This may be explained by the multifactorial nature of cancer-related immune alterations, which vary depending on individual patient factors, tumor biology, and treatment responses. This suggests that global immune profiling alone may not be sufficient to stratify patients and that targeted biomarker analysis is necessary to uncover clinically relevant patterns.

One of the key findings in our study is the dual anti-inflammatory and anabolic effect of ONS enriched with leucine and EPA/DHA compared to standard ONS. Patients receiving enriched ONS exhibited lower levels of inflammatory markers such as TRAIL and LAMP3, two mediators associated with systemic inflammation, and increased levels of galectin-1 (al-1), a protein involved in muscle regeneration. TRAIL is released by neutrophils in response to pro-inflammatory stimuli, such as interleukin-8 (IL-8) and tumor necrosis factor alpha (TNF-α), and it is known to exhibit both pro-inflammatory and pro-apoptotic properties. Although we did not observe differences in IL-8 or TNF-α levels, the increase in TRAIL expression suggests that patients treated with standard ONS experience a higher degree of inflammation than those treated with enriched ONS. Therefore, such a formula might prevent an increase in inflammation in these patients and thereby reduce muscle catabolism [9]. In addition, increased serum TRAIL levels have been shown to predict severity and correlate with poorer prognosis in diseases like pneumonia [10].

Similarly, LAMP3 remained higher in the standard ONS group, further supporting the anti-inflammatory effect of the enriched formulation. Although its specific function has not been identified yet, LAMP3 has been considered a biomarker of poor prognosis in tissue from esophageal squamous cell carcinoma patients [11]. In addition, LAMP3 overexpression in tissue promotes metastasis of breast cancer cells [12,13], while its increased expression is associated with poor overall survival in patients with gastrointestinal cancer, cervical cancer, and breast cancer [12,14,15].

Elevated levels of Gal-1 were observed in patients receiving enriched ONS. Gal 1 has been shown to enhance muscle regeneration, promoting muscle growth and repair [16]. In addition, Gal-1 has been shown to be involved with improved muscle function and sarcolemma integrity in murine models of muscular dystrophy, confirming that Gal-1 contributes to improved body composition in patients receiving enriched ONS, increasing the anabolic status of muscle mass [17,18]. Hence, Gal-1 has emerged as a crucial player in myogenic differentiation, with its increased levels in patients who gained muscle mass highlighting the potential of the enriched ONS formula to augment muscle regeneration. These findings suggest that enriched ONS may promote muscle repair and growth by influencing intracellular signaling pathways associated with Gal-1 and other key proteins. The ONS formula enriched with leucine and EPA/DHA is a promising intervention for preserving muscle mass and function in cancer patients, particularly those at risk of sarcopenia.

Given that patients treated with enriched ONS displayed an increase in their muscle mass [4] and that TRAIL (which also reduces muscle catabolism) was decreased in these patients while Gal-1 (which enhances muscle function) was increased after the intervention, to further explore the variability in treatment response, we stratified patients based on muscle mass outcomes before performing the ONS intervention in order to determine whether we could predict its outcome. Our results reveled that patients who did not gain muscle mass had higher soluble levels of mucin 16 (MUC16), arginase 1 (ARG1), and interleukin 12 receptor beta 1 subunit (IL12RB1) (three mediators associated with tumor progression, immune suppression, and chronic inflammation) compared to their counterparts from the standard ONS group (Figure 3). MUC16 is increased in multiple cancer types, playing important roles in their tumorigenicity [19,20]. In a similar manner, ARG1 plays a pivotal role in tumorigenesis and metastasis, and it can be used as potential diagnosis biomarker for cancer progression [21,22], while the soluble form of IL12RB1 may truncate the capacity of the immune system to trigger a pro-inflammatory Th1/Th17 response [23]. Together, these results suggest that patients who did not gain muscle mass after treatment with enriched ONS had more aggressive molecular inflammation, hence providing a specific mechanism of action to explain the loss in muscle mass despite the enriched ONS. Moreover, the identification of MUC16, ARG1, and IL12RB1 as potential biomarkers to predict muscle gain before starting enriched ONS treatment may therefore allow for the development of personalized interventions.

Having proven the presence of immune differences between patients who gained muscle mass and those who did not, we finally assessed the dynamics of these mediators based on the muscle gain/loss outcome. Our results revealed that the patients who lost muscle mass after enriched ONS treatment had higher levels of soluble placenta growth factor (PGF), CD28, and beta 1 receptor for IL12/23 (IL12RB1), even before the intervention, compared to patients who gained muscle mass. Although it has been shown that PFG stimulates muscle growth [24], it was nevertheless increased in the patients who did not respond to treatment, maybe as a compensatory mechanism. In addition, its levels increased overtime in the patients who gained muscle mass following the enriched ONS treatment. CD28 is a regulator of glucose metabolism [25] and a key player in various immune-related diseases, such as asthma [26,27], autoimmune diseases like lupus [28], and Graves’ disease [29]. Elevated soluble CD28 levels are also associated with inflammation associated with immune senescence, or inflammaging, a process characterized by chronic inflammation that cannot be resolved [30]. Hence, the patients who did not gain muscle mass may therefore have displayed a more inflamed basal state. Soluble IL12RB1 is directly related to sarcopenia [31]. The patients who received enriched ONS who lost muscle mass had a higher expression of this receptor compared to those who gained muscle mass before the treatment. After the treatment, and although not significant, there was a trend showing that the patients who gained muscle mass had a lower expression of this receptor. When looking at IL12 itself, no significant differences were observed between patients, although the pattern was similar. Moreover, the higher baseline levels of PGF, CD28, and IL12RB1 in the patients who lost muscle mass before the intervention suggest that these biomarkers could be used as early predictors of treatment response.

Our results therefore underscore the immunonutritional properties of the ONS formula enriched with leucine and EPA/DHA, which significantly influences key mediators involved in muscle differentiation and the inflammatory status of patients with cancer and DRM. The observed modulation of proteins such as TRAIL, LAMP3, GAL1, MUC16, ARG1, IL12RB1, PGF, and CD28 suggests, therefore, that the enriched ONS formula not only supports muscle mass gain but also helps to mitigate inflammation, a critical factor in muscle health. Of note is the fact that since no differences were found in any immune or oncology mediators between the patients with an increase or decrease in their muscle mass treated with the standard ONS, our findings confirm that the enriched formula has immunomodulatory properties, hence providing a mechanism by which it elicits its effects in terms of the gain in the muscle mass, thereby revealing novel biomarkers that can predict such an outcome.

We are nevertheless aware that further research is needed to fully elucidate the mechanisms by which enriched ONS treatment influences the expression of these proteins and its subsequent impact on muscle mass and overall muscle health. Understanding these mechanisms could lead to the development of new nutritional strategies aimed at enhancing muscle regeneration and combating muscle-wasting conditions, offering significant benefits for patients suffering from muscle degeneration and chronic inflammation. In a similar manner, we are also aware of the restricted sample size in this analysis (which did not include any sample calculation size), which was due to this being a pilot study. Nevertheless, it is also true that the obtained results provide a biological meaning and explanation for the observed outcomes in the patients. Hence, our group will engage in future prospective and independent studies that take into account specific parameters, as well as a relevant variation in terms of the calculation of an appropriate sample size, in order to confirm the results obtained here.

In summary, we hereby provide a mechanism by which enriched ONS elicits its effect on muscle mass gain in cancer patients while also identifying novel biomarkers that predict treatment outcomes. This, therefore, may lay the basis for performing precision medicine in emerging areas like immunonutrition in the oncology field.

## 5. Strengths and Limitations

This study presents several strengths and limitations that should be acknowledged. Among its main strengths is the novel integration of soluble oncoimmunome profiling with clinical and body composition outcomes, providing a mechanistic insight into how enriched immunonutrition may modulate inflammatory pathways and promote muscle mass accretion in cancer patients with disease-related malnutrition. The use of a randomized, double-blind, controlled design alongside standardized nutritional and immunological assessments adds methodological robustness. Moreover, the identification of predictive immune biomarkers before the intervention supports the feasibility of precision nutrition approaches in clinical oncology. However, certain limitations must be considered. The sample size was relatively small and may limit the generalizability of the findings, especially in diverse cancer types and stages. In addition, although the proteomic analysis was comprehensive, it focused solely on circulating soluble markers without evaluating tissue-level immune responses or functional outcomes such as strength or quality of life. As this was a subanalysis, the study was not originally tailored to detect immunological differences as primary outcomes. Future large-scale, multicenter studies are warranted to validate the predictive value of the identified biomarkers and to confirm the reproducibility of the immunomodulatory effects observed with enriched ONS formulations

## 6. Conclusions

In conclusion, our findings underscore the potential of enriched oral nutritional supplementation (ONS), not only as a means to improve caloric and protein intake but also as a targeted immunonutritional strategy capable of modulating systemic inflammation and enhancing muscle mass in cancer patients with disease-related malnutrition. The identification of specific soluble immune biomarkers predictive of treatment response—such as IL12RB1, PGF, and galectin-1—offers a promising pathway towards the personalization of nutritional care. These insights support the integration of immune profiling into clinical decision-making, allowing for more precise and effective nutritional interventions tailored to the inflammatory and metabolic status of each patient. Future research should validate these biomarkers in larger cohorts and explore their applicability across different oncological and chronic inflammatory conditions. Ultimately, this approach may contribute to optimizing therapeutic outcomes, preserving functional status, and improving quality of life in vulnerable patient populations.

## Figures and Tables

**Table 1:** Compositions of macronutrients and ingredients of the formulas under study per 100 mL.

	Enriched ONS (A)	Standard ONS (B)
Energy (kcal)	200	200
Proteins, g/TE%, (ingredients) L-leucine	10 g/20% [whey protein (63%), caseinate, and vegetal protein] 1.6 g	10 g/20% [caseinate and whey protein (25%)] 0.9 g
Carbohydrates: g/TE%, (ingredients) Sugars	20 g/39.7% (dextrin and maltodextrin) 2.4 g	18.2 g/46% (maltodextrin) 2.1 g
Fat g/TE% (ingredients)	8.6 g/38.5% (EVOO, canola, MCT, and fish oil)	5 g/(31%) (canola and high-oleic sunflower oil)
SFA MUFA PUFA EPA and DHA	10.9% 18.6% 9.0% 750 mg	4.4% 19.1% 6.5% -
Fiber g/TE% (ingredients)	1.8 g/1.8% (FOS, acacia fiber and beta glucans) 100% soluble	1.5 g/1.5%(FOS and oat fiber) 60% soluble–40% insoluble
Osmolarity (mOsm/L)	420	390
Flavor	Natural vanilla	Natural vanilla
Sweetener	Sucralose	Sucralose

EVOO: extra-virgin olive oil; FOS: fructooligosaccharides; MCT: medium-chain triglyceride; MUFA: monounsaturated fatty acid; Enriched ONS: enriched oral nutritional supplementation; Standard ONS: standard oral nutritional supplementation; PUFA: polyunsaturated fatty acid; TE%: percentage of total energy. A: Bi1 Alisenoc^®^, Nutrimed Clinical Nutrition, Spain. B: Bi1 Control 2.0^®^, Nutrimed Clinical Nutrition, Spain.

**Table 2:** Caloric and protein intakes before and after nutritional interventions.

**Standard ONS**	**Unit**	**Before**	**After**
Energy intake	kcal	1309.75 (372.18)	2147.48 (347.28) ***
Protein	g	55.78 (16.76)	96.25 (13.19) ***
Leucine	g	0.7 (0.6)	1.3 (0.7)
Carbohydrate	g	126.78 (42.72)	221.06 (40.32) ***
Fat	g	61.34 (23.82)	92.12 (23.07) *
SFA	g	18.1 (7.62)	25.22 (6.27) *
MUFA	g	28.43 (11.54)	43.57 (12.72) **
PUFA	g	6.94 (3.15)	13.88 (4.39) ***
EPA and DHA	g	0.5 (0.3)	0.7 (0.4)
Fiber	g	13.56 (8.18)	19.25 (9.28) ***
Energy per body weight	kcal/kg	24.68 (8.65)	39.82 (9.57) ***
Protein per body weight	g/kg	1.07 (0.45)	1.78 (0.36) ***
**Enriched ONS**	**Units**	**Before**	**After**
Energy intake	kcal	1459.58 (276.37)	2222.01 (470.05) ***
Protein	g	61.03 (13.71)	101.91 (20.03) ***
Leucine	g	0.6 (0.4)	3.3 (0.9) **
Carbohydrate	g	137.76 (35.77)	210.91 (51.14) ***
Fat	g	71.18 (18.98)	102.98 (24.84) **
SFA	g	22.42 (8.34)	29.68 (8.50) *
MUFA	g	34.14 (10.34)	49.69 (13.36) *
PUFA	g	7.57 (2.14)	15.97 (3.65) ***
EPA and DHA	g	0.4 (0.2)	1.4 (0.3) **
Fiber	g	11.79 (4.64)	20.92 (6.76) ***
Energy per body weight	kcal/kg	25.78 (8.62)	37.16 (8.73) **
Protein per body weight	g/kg	1.09 (0.45)	1.70 (0.37) ***

SFA: saturated fatty acid; MUFA: monounsaturated fatty acid; EPA: eicospentaenoic acid; DHA: docosahexanoic acid; Enriched ONS: enriched oral nutritional supplementation; Standard ONS: standard oral nutritional supplementation; PUFA: polyunsaturated fatty acid. A paired *t*-test was applied to compare the levels before and after the intervention (* *p* < 0.05; ** *p* < 0.01; *** *p* < 0.001), while a classical *t*-test was applied to compared levels among groups at the end of the intervention (*p* < 0.05).

**Table 3:** Clinical characteristics of patients in both groups.

	Standard (n = 14)		Enriched (n = 14)	
	Before	After	Before	After
Body weight (kg)	61.7 ± 13.1	61.4 ± 12.3	67.3 ± 17.1	68.7 ± 6.1 *
Muscle mass (kg)	19.6 ± 7.1	18.7 ± 6.2	18.5 ± 8.0	20.7 ± 8.5 *
BMI (kg/m^2^)	23.1 ± 4.1	23.0 ± 3.6	24.5 ± 5.6	25.1 ± 6.1

Body weight, muscle mass, and body mass index (BMI) from patients undergoing both the standard and the enriched oral nutritional supplementation are shown before and after the intervention. A paired *t*-test was applied within each group. A *p*-value < 0.05 was considered statistically significant (* *p* < 0.05).

**Table 4:** Soluble immunome before and after the interventions.

	Enriched ONS (A) (n = 14)		Standard ONS (B) (n = 14)	
	Before	After	Before	After
**IL8**	8.46 ± 1.52	8.23 ± 1.51	8.64 ± 1.98	7.6 ± 1.05
**TNFRSF9**	6.11 ± 0.96	5.98 ± 0.75	5.81 ± 0.71	5.92 ± 0.81
**TIE2**	6.96 ± 0.35	7.05 ± 0.29	7.08 ± 0.24	7.09 ± 0.27
**MCP_3**	3.95 ± 1.6	3.76 ± 0.78	3.71 ± 0.95	3.53 ± 1.22
**CD40_L**	7.93 ± 1.71	8.49 ± 0.77	8.18 ± 1.31	7.69 ± 1.63
**IL_1alpha**	0.75 ± 0.53	1.18 ± 1.01	0.85 ± 0.4	0.69 ± 0.36
**CD244**	6.63 ± 0.47	6.62 ± 0.32	6.43 ± 0.34	6.56 ± 0.64
**EGF**	9.35 ± 1.52	9.74 ± 0.69	9.3 ± 1.31	9 ± 1.33
**ANGPT1**	7.06 ± 0.86	7.33 ± 0.16	7.11 ± 0.88	7.18 ± 0.26
**IL7**	5.06 ± 1.37	5.32 ± 1.08	5.38 ± 1.09	5.44 ± 0.99
**PGF**	9.25 ± 0.75	9.28 ± 0.43	8.92 ± 0.52	8.94 ± 0.46
**IL6**	4.83 ± 1.7	4.36 ± 1.76	5.35 ± 2	4.54 ± 1.78
**ADGRG1**	2.05 ± 1.31	2.1 ± 1.2	2.02 ± 1.4	2.04 ± 1.01
**MCP_1**	12.63 ± 0.86	12.82 ± 0.44	12.63 ± 0.72	12.47 ± 0.73
**CRTAM**	5.84 ± 1	5.65 ± 0.75	5.57 ± 0.8	5.43 ± 0.73
**CXCL11**	9.3 ± 1.27	9.59 ± 1.23	9.61 ± 1.32	9.34 ± 1.21
**MCP_4**	10.79 ± 0.97	11.08 ± 0.57	10.63 ± 0.82	10.62 ± 0.78
**TRAIL**	8.5 ± 0.44	**8.51 ± 0.4** *	8.72 ± 0.29	8.78 ± 0.22
**FGF2**	0.94 ± 0.75	1.12 ± 0.43	0.99 ± 1.11	1.03 ± 1.24
**CXCL9**	8.28 ± 1.28	8.26 ± 0.83	8.18 ± 1.08	8.09 ± 1
**CD8A**	9.63 ± 0.64	9.48 ± 0.54	9.37 ± 0.63	9.31 ± 0.93
**CAIX**	5.2 ± 1.12	4.86 ± 0.84	5.18 ± 0.81	5.39 ± 0.97
**MUC_16**	5.67 ± 1.47	5.26 ± 1.56	5.49 ± 1.2	5.23 ± 1.38
**ADA**	6.11 ± 0.58	5.93 ± 0.54	6 ± 0.63	6.06 ± 0.53
**CD4**	4.09 ± 0.53	4.05 ± 0.41	3.98 ± 0.39	3.93 ± 0.36
**NOS3**	2.71 ± 0.83	2.61 ± 0.45	2.61 ± 0.66	2.53 ± 0.9
**IL2**	0.57 ± 0.79	0.72 ± 0.56	0.94 ± 0.39	0.63 ± 0.38
**Gal_9**	9.09 ± 0.56	9.08 ± 0.43	8.98 ± 0.44	9.03 ± 0.33
**VEGFR_2**	8.55 ± 0.46	8.63 ± 0.29	8.6 ± 0.27	8.56 ± 0.25
**CD40**	11.83 ± 0.73	11.84 ± 0.5	11.58 ± 0.58	11.62 ± 0.77
**IL18**	9.5 ± 0.64	9.55 ± 0.86	9.63 ± 0.89	9.75 ± 0.8
**GZMH**	4.74 ± 1.25	4.41 ± 0.67	4.52 ± 1.31	4.32 ± 1.01
**KIR3DL1**	2.23 ± 1.64	2.15 ± 1.58	2.27 ± 0.98	2.35 ± 1.13
**LAPTGF_beta_1**	8.74 ± 0.66	8.82 ± 0.45	8.8 ± 0.67	8.77 ± 0.6
**CXCL1**	9.39 ± 0.88	9.58 ± 0.75	9.73 ± 0.75	9.31 ± 0.94
**TNFSF14**	6.33 ± 1.34	6.29 ± 1.07	6.62 ± 1.34	6.08 ± 1.04
**IL33**	0.44 ± 0.24	0.44 ± 0.34	0.39 ± 0.2	0.34 ± 0.22
**TWEAK**	9.13 ± 0.62	9.21 ± 0.3	9.09 ± 0.43	9.06 ± 0.45
**PDGFsubunitB**	10.56 ± 0.56	10.82 ± 0.11	10.64 ± 0.47	10.67 ± 0.28
**PDCD1**	8.1 ± 1.3	8.3 ± 1.11	8.06 ± 1.3	7.86 ± 1.04
**FASLG**	7.33 ± 1.16	7.32 ± 0.7	7.56 ± 0.67	7.37 ± 0.97
**CD28**	0.31 ± 0.57	0.37 ± 0.35	0.33 ± 0.38	0.35 ± 0.46
**CCL19**	11.67 ± 0.92	11.44 ± 0.8	11.48 ± 0.59	11.36 ± 0.61
**MCP_2**	8.91 ± 0.95	9 ± 0.51	8.64 ± 0.56	8.61 ± 0.6
**CCL4**	7.68 ± 0.78	7.62 ± 1.02	7.71 ± 1.03	7.11 ± 0.68
**IL15**	5.65 ± 0.47	5.66 ± 0.36	5.89 ± 0.62	5.77 ± 0.45
**Gal_1**	8.05 ± 0.4	**8.14 ± 0.29** *	7.85 ± 0.31	7.86 ± 0.3
**PD_L1**	7.15 ± 0.61	7.1 ± 0.44	7.15 ± 0.62	7.14 ± 0.53
**CD27**	9.36 ± 0.69	9.33 ± 0.57	9.23 ± 0.56	9.16 ± 0.51
**CXCL5**	9.72 ± 1.09	10.09 ± 0.64	9.76 ± 0.69	9.53 ± 0.95
**IL5**	1.27 ± 0.85	1.2 ± 0.75	1.1 ± 0.76	1.17 ± 0.54
**HGF**	10.08 ± 0.8	10.09 ± 0.61	10.32 ± 0.86	10.01 ± 0.59
**GZMA**	7.12 ± 0.79	6.9 ± 0.49	6.92 ± 0.65	6.79 ± 0.65
**HO_1**	12.76 ± 0.5	12.64 ± 0.35	12.84 ± 0.38	12.84 ± 0.3
**CX3CL1**	4.59 ± 0.74	4.56 ± 0.53	4.55 ± 0.45	4.54 ± 0.46
**CXCL10**	9.54 ± 1.06	9.63 ± 0.84	9.28 ± 0.95	9.2 ± 0.89
**CD70**	5.09 ± 0.82	5.05 ± 0.48	4.93 ± 0.74	5.04 ± 0.65
**IL10**	3.74 ± 0.99	3.42 ± 0.71	3.71 ± 1.13	3.29 ± 0.81
**TNFRSF12A**	8.89 ± 0.77	8.92 ± 0.53	8.55 ± 0.78	8.59 ± 0.55
**CCL23**	12.6 ± 0.61	12.48 ± 0.6	12.39 ± 0.65	12.3 ± 0.6
**CD5**	5.73 ± 0.72	5.64 ± 0.49	5.65 ± 0.55	5.54 ± 0.43
**CCL3**	8.33 ± 1.57	7.93 ± 1.58	8.14 ± 1.73	7.33 ± 0.82
**MMP7**	12.61 ± 1.04	12.89 ± 0.39	12.27 ± 1.49	12.55 ± 0.68
**ARG1**	4.8 ± 1.17	4.94 ± 1.18	5.19 ± 1.1	4.69 ± 0.75
**NCR1**	4.49 ± 0.81	4.61 ± 0.69	4.41 ± 0.68	4.47 ± 0.65
**DCN**	4.63 ± 0.49	4.6 ± 0.28	4.49 ± 0.37	4.55 ± 0.39
**TNFRSF21**	10.75 ± 0.28	10.8 ± 0.21	10.73 ± 0.26	10.74 ± 0.22
**TNFRSF4**	8.22 ± 0.83	8.13 ± 0.6	7.9 ± 0.62	7.98 ± 0.53
**MIC_A/B**	5.94 ± 2.15	6 ± 2.01	6.13 ± 2.02	6.05 ± 1.9
**CCL17**	10.72 ± 1.19	11.25 ± 0.72	10.8 ± 1.09	10.79 ± 0.84
**ANGPT2**	6.14 ± 0.74	6.05 ± 0.66	5.87 ± 0.73	5.83 ± 0.41
**PTN**	1.69 ± 0.62	1.78 ± 0.57	1.41 ± 0.65	1.8 ± 0.59
**CXCL12**	1.55 ± 0.5	1.54 ± 0.33	1.51 ± 0.3	1.49 ± 0.51
**IFN_gamma**	7.98 ± 1.24	8.15 ± 0.97	8.9 ± 1.99	8.71 ± 1.61
**LAMP3**	4.54 ± 0.65	4.63 ± 0.72 *	4.98 ± 0.74	5.24 ± 0.82
**CASP_8**	6.64 ± 0.95	6.57 ± 1.17	6.46 ± 1.05	6.39 ± 1.35
**ICOSLG**	6.19 ± 0.61	6.45 ± 0.77	6.01 ± 0.38	6.04 ± 0.36
**MMP12**	8.8 ± 0.83	8.45 ± 0.65	8.69 ± 1.45	8.26 ± 0.58
**CXCL13**	9.67 ± 1.04	9.43 ± 0.7	9.55 ± 0.93	9.61 ± 0.89
**PD_L2**	3.25 ± 0.6	3.2 ± 0.54	3.14 ± 0.55	3.2 ± 0.57
**VEGFA**	10.37 ± 0.98	10.37 ± 0.88	10.58 ± 1.02	10.35 ± 0.73
**IL4**	1.2 ± 0.54	0.88 ± 0.58	1.13 ± 0.37	0.81 ± 0.58
**LAG3**	4.93 ± 0.77	5.19 ± 0.51	4.98 ± 0.8	4.96 ± 0.76
**IL12RB1**	3.07 ± 0.84	2.96 ± 0.7	2.86 ± 0.53	2.88 ± 0.5
**IL13**	0.31 ± 1.15	0.18 ± 0.57	0.66 ± 0.65	0.41 ± 0.68
**CCL20**	8.52 ± 1.35	7.89 ± 1.23	8.51 ± 1.95	8.25 ± 1.75
**TNF**	4.95 ± 1.35	4.71 ± 1.61	4.85 ± 1.31	4.2 ± 0.79
**KLRD1**	7.69 ± 0.85	7.61 ± 0.68	7.22 ± 0.87	7.25 ± 0.86
**GZMB**	5.28 ± 0.96	4.88 ± 0.65	5.14 ± 1.1	4.89 ± 0.78
**CD83**	2.87 ± 0.76	2.88 ± 0.57	2.93 ± 0.54	2.92 ± 0.46
**IL12**	5.37 ± 1.69	5.73 ± 1.07	5.38 ± 1.24	5.34 ± 1.31
**CSF_1**	10.06 ± 0.32	10.01 ± 0.19	10.04 ± 0.31	9.98 ± 0.24

Mean levels and standard deviation (in arbitrary units) of each soluble immune mediator both before and after standard or enriched oral nutritional supplementation (ONS). **A: Enriched ONS; Bi1 Alisenoc^®^, Nutrimed Clinical Nutrition,** Spain; **B: Standard ONS; Bi1 Control 2.0**^®^, **Nutrimed Clinical Nutrition**, Spain. * Asterisks indicate statistically significant differences between the standard ONS and enriched ONS groups at the post-intervention timepoint (*p* < 0.05). All other significant comparisons are described in the Results section.

## Data Availability

All data generated or analyzed during this study are included in this article. Further enquiries can be directed to the corresponding authors.
